# Professional Advertising

**Published:** 1883-12

**Authors:** 


### Professional Advertising.

The Journal has received a communication from the Lecturer on Surgery at the Buffalo Medical College, disclaiming all responsibility for the objectionable advertising which drew forth our just stricture in the last number of the Journal.

We are glad to learn that the authorities of the Buffalo Medical College have informed the students that they will not be permitted to report cases for the secular press.

We hope, therefore, that the profession of the city will not be again scandalized by the publication of highly colored reports of operations, etc., with the plain motive of parading the name of the surgeon before the community and thus striving to establish a reputation by objectionable devices. Our duty as journalists required us to censure such violation of the ethics of the profession with severity, but in doing so we disclaim any animosity or ill feeling towards the surgeon whom it was thus proposed to benefit. It was to us an unpleasant incident in our controversy with the college relative to the appointment of a surgeon to the hospital, that in our protest against the ignoring of the just claims of the surgeons of our own city, we were compelled to assume a position which has been, by some, construed as antagonistic to the new-comer.

We are heartily glad, therefore, to be able to publish his disclaimer as above, and we hope he will not further suffer from the ill-advised efforts of his friends to aid him in the attainment of professional reputation.

The authorities of the Buffalo Medical College also disclaim responsibility, asserting that they were unable to control their students. If this is so we repeat what we said in our last: it is a pitiful evidence of weakness and inefficiency. We believe, however, that they underrate their own abilities; but in any case, the profession will hold them responsible for such gross violation of professional ethics, as the publication in the daily papers of sensational reports of cases occurring in the clinics of the college and hospital, over which they are supposed to have control.

